# Rutaecarpine alleviates hepatic ischemia‒reperfusion injury in liver transplantation by inhibiting inflammatory response and oxidative stress

**DOI:** 10.3389/fphar.2025.1539744

**Published:** 2025-02-03

**Authors:** Yan Liu, Feng Qi, Lun-Jian Xiang, Zhu-Jun Yi, Sheng-Wei Li

**Affiliations:** ^1^ Department of Hepatobiliary Surgery, The Second Affiliated Hospital of Chongqing Medical University, Chongqing, China; ^2^ Department of Hepatobiliary Surgery, Chongqing University Three Gorges Hospital, Chongqing, China

**Keywords:** donation after circulatory death, liver transplantation, rutaecarpine, oxidative stress, inflammatory response

## Abstract

**Background:**

Donation after circulatory death (DCD) livers are limited by mandatory warm ischemia and are more susceptible to ischemia‒reperfusion injury (IRI). Inflammation and oxidative stress play key roles in the development of hepatic IRI, and Rutaecarpine (Rut) has anti-inflammatory and anti-oxidative stress effects. The aim of this study was to investigate whether Rut can alleviate hepatic IRI in liver transplantation (LT) and to explore the underlying mechanisms.

**Methods:**

Rat DCD LT and oxygen-glucose deprivation/reoxygenation (OGD/R) cell models were established to clarify the effect of Rut on hepatic IRI. The key molecules involved in the hepatoprotective effects of Rut were identified through joint analysis of data from LT patients and drug targets. The target was further validated by *in silico*, *in vivo* and *in vitro* experiments.

**Results:**

Rut significantly alleviated liver dysfunction, pathological injury, and apoptosis and improved the survival rate of the rats subjected to LT. In addition, Rut significantly inhibited inflammatory response and oxidative stress. Rut also had similar effects on OGD/R-induced hepatocyte injury. Mechanistically, bioinformatics analysis and *in vivo* and *in vitro* experiments revealed that PDE4B may be a key target by which Rut exerts its protective effect, and molecular docking and cellular thermal shift assay confirmed this result. The function of PDE4B was studied *via* gene intervention technology, and the results showed that PDE4B can aggravate hepatic IRI. Furthermore, PDE4B overexpression abrogated the protective effect of Rut on the liver in LT.

**Conclusion:**

Rut alleviates hepatic IRI by targeting PDE4B to inhibit inflammation and oxidative stress. These findings highlight the potential of Rut as a drug candidate for the treatment of patients undergoing LT.

## 1 Introduction

Liver transplantation (LT) can bring new life to patients with end-stage liver disease, but only approximately 40% of patients on the waiting list have access to liver transplantation (LT) within 1 year, and 12% of candidates die due to organ shortages (Schwab et al., 2024). Donation after circulatory death (DCD) can mitigate the problem of organ shortages to some extent. However, because the DCD liver needs to experience fixed warm ischemia, it is extremely sensitive to IRI and has poor tolerance (Hashimoto, 2020; Meier et al., 2023). IRI will lead to primary nonfunction and early allograft dysfunction, which seriously affects the prognosis of patients (Masior and Grat, 2022; Zhai et al., 2013). Currently, the drugs and methods used for IRI have not yet achieved satisfactory results. Therefore, we still need to actively look for effective drugs or interventions.

Accumulating evidence suggests that traditional Chinese medicine monomers have great therapeutic potential in relieving hepatic IRI. We previously reported that wogonin relieves hepatic IRI through the inhibition of ferroptosis and that oridonin relieves hepatic IRI by inhibiting macrophage pyroptosis (Jia et al., 2024; Wu et al., 2024). However, owing to their high hydrophobicity, wogomin and oridonin have low solubilities and poor bioavailabilities (Yang et al., 2022; Zhang et al., 2020). Rutaecarpine (Rut) is a quinazolinocarboline alkaloid extracted from Evodia rutaecarpa, and Evodia rutaecarpa has been used to treat various inflammation-related diseases, including headache and gastrointestinal diseases (Kshirsagar, 2015). Although Rut is also rather hydrophobic, it has a high hepatic extraction ratio (Estari et al., 2021; Ko et al., 1994). As the most important active ingredient of Evodia rutaecarpa, Rut has strong anti-inflammatory effects. For example, cyclooxygenase-2 is a key determinant of inflammation, and Rut can act as a cyclooxygenase-2 inhibitor to relieve rat lambda-carrageenan-induced paw edema (Chun et al., 2024; Moon et al., 1999). In addition, Rut can relieve t-BHP-induced hepatotoxicity by activating the Nrf-2-mediated antioxidant system and relieve hypoxia-reoxygenation-induced myocardial cell apoptosis by the inhibition of the NADPH oxidase-ROS pathway (Bao et al., 2011; Jin et al., 2017). Although the pathogenesis of IRI is not fully understood, a large amount of evidence suggests that inflammation and oxidative stress are critical in the development of IRI (Dar et al., 2019). Therefore, Rut may be applicable for LT. Nevertheless, previous studies have not focused on this pathological process to explore the role of Rut.

In this study, we aimed to establish rat DCD LT and hepatocyte OGD/R models to clarify the function of Rut in hepatic IRI and explore the underlying mechanisms.

## 2 Materials and methods

### 2.1 Reagents used

Rut (CAS84-26-4) was purchased from Aladdin Reagent Co., Ltd (Shanghai, China), the specific antibody for IL-6 used in this study was purchased from Abcam (Cambridge, UK), and the specific antibody for phosphodiesterase 4B (PDE4B) was purchased from Novus Biologicals, Inc. The protein assay kit was purchased from Beyotime Biotechnology (Jiangsu, China). A hematoxylin and eosin (H&E) staining kit was provided by Solarbio (Beijing, China). Lipofectamine 3000 transfection reagent was purchased from Invitrogen Co., Ltd (California, United States). Malondialdehyde (MDA) and catalase (CAT) detection kits were purchased from Nanjing Jiancheng Bioengineering Institute (China, Jiangsu), and glutathione (GSH) and superoxide dismutase (SOD) detection kits were purchased from Servicebio Biotechnology (China, Hubei).

### 2.2 Animals

In this study, healthy Sprague‒Dawley (SD) rats (seven to eight weeks, 250–280 g) were used as experimental animals. All animals were bred under standard conditions and had free access to food and water. The animal experiments in this study were approved by the Animal Use and Ethics Committee of the Second Affiliated Hospital of Chongqing Medical University (IACUC-SAHCQMU-2023-0008). We performed the animal studies strictly according to the ARRIVE guidelines. We tried our best to relieve the pain and distress of the animals.

### 2.3 DCD rat LT model and drug injection

The DCD rat LT model was established according to our previous study (Jia et al., 2024; Liu et al., 2024). Briefly, DCD was simulated by inducing suffocation. The warm ischemia time was defined as the interval between the cessation of pulsatile blood pressure in the rat and the initiation of cold perfusion, with the duration controlled at 45 min. The infrahepatic inferior vena cava and the portal vein were anastomosed using cuff, and magnetic rings were used for suprahepatic inferior vena cava anastomosis. The anhepatic phase was strictly limited to 15 min, during which the portal vein will not be opened. All surgeries were completed under inhalation anesthesia.

The rats were randomly divided into six groups *via* the random number table method: the sham group, the Rut group, the LT group, and the LT + Rut (30 mg/kg, 60 mg/kg, 120 mg/kg) group. There were six rats in the sham group and Rut group and six pairs (12) of rats in each other group for the following experiments. An additional seven pairs (14 rats) were used in the LT and LT + Rut (60 mg/kg) groups to observe the 7-day survival rate. The corresponding doses of Rut were injected intraperitoneally into the recipients 30 min before surgery. To reduce potential bias, the administration of Rut or solvents to the rats was performed by a single person, and the surgeon was unaware of the grouping of the rats. Blood and liver samples were collected 6 h after reperfusion for subsequent detection.

### 2.4 Liver function tests

Blood samples were centrifuged to separate serum. The levels of alanine aminotransferase (ALT) and aspartate aminotransferase (AST) in the serum were measured *via* an automatic biochemical analyzer (Rayto, China).

### 2.5 OGD/R cell model and drug treatment

The rat hepatocytes (BRL-3A) used in this study were purchased from Procell Biological Co., Ltd (Hubei, China). The cells were incubated in DMEM/F12 medium (Gibco) containing 10% FBS (Gibco, United States) under 37°C in a 5% CO_2_ environment. The oxygen-glucose deprivation/reoxygenation (OGD/R) cell model was constructed as described previously (Jia et al., 2024). Briefly, the cells were first cultured in RPMI-1640 medium for 6 h in a three-gas incubator (1% O_2_, 5% CO_2_, 94% N_2_, 37°C) and then changed to DMEM/F12 medium and incubated in a normal incubator (5% CO_2_, 37°C) for 12 h. Rut was used to pretreat cells for examining its protective effect.

### 2.6 Detection of cell viability

Cell viability was determined *via* the CCK8 assay. The cells were seeded in 96-well plates. When the cells grew to an appropriate density, Rut was added for pretreatment, and then the cells were subjected to OGD/R. CCK8 reagent was added, the mixture was incubated for 3 h, and cell viability was measured by measuring the absorbance *via* a microplate reader.

### 2.7 Protein extraction and western blot

Protein extraction from cell and tissue samples was performed *via* RIPA lysis buffer. Proteins were subjected to SDS‒PAGE and transferred to a PVDF membrane, which was blocked in 5% skim milk for 1 h. Then, the PVDF membrane was incubated with PDE4B- and GAPDH-specific primary antibodies at 4°C overnight. Next day, the PVDF membrane was incubated with the corresponding specific secondary antibodies at room temperature. The membrane was subsequently incubated with chemiluminescence reagents and photographed in a Bio-Rad gel imaging system.

### 2.8 Pathology and immunohistochemistry of the liver

After fixation and paraffin-embedding, liver tissue samples were sectioned into 4–5 μm thick sections and subjected to H&E staining to observe morphological changes in the tissue. Immunohistochemistry was performed using universal staining kit (PV-6000, ZSGB-BIO) according to the manufacturer’s instructions. The sections were co-incubated with an IL-6-specific primary antibody and a horseradish peroxidase-labeled IgG polymer. The sections were then developed with DAB and observed under a microscope.

### 2.9 Gene silencing and overexpression

Specific gene silencing was achieved *via* siRNA technology, and gene overexpression was achieved *via* plasmid transfection. siRNAs and plasmids (Sangon Biotechnology, China) were transfected into BRL-3A cells using Lipofectamine 3000 transfection reagent according to the manufacturer’s instructions.

### 2.10 RNA extraction and RT‒PCR

Total RNA from animals or cells was extracted *via* the TRIzol method. The concentration and purity of the RNA were detected *via* a NanoDrop detection analyzer. The cDNA synthesis was performed *via* a two-step method with gDNA Clean Reaction Mix and RT Reaction Mix (Accurate Biology). Real-time PCR (RT‒PCR) was performed using SYBR Green Pro Taq HS Premix IV (Accurate Biology). The sequences of primers used in this study are listed in Table 1 **.**

**TABLE 1:** Primer sequences for RT‒PCR.

Gene name	Primer sequence	
	Forward (5′− 3′)	Reverse (5′− 3′)
TNF-α	TCA​GTT​CCA​TGG​CCC​AGA​C	GTT​GTC​TTT​GAG​ATC​CAT​GCC​ATT
IL-1β	AAG​CTC​TCC​ACC​TCA​ATG​GAC	GTG​CCG​TCT​TTC​ATC​ACA​CAG
IL-6	AGC​CAG​AGT​CAT​TCA​GAG​CA	TGG​TCT​TGG​TCC​TTA​GCC​AC
IL-10	GCA​GTG​GAG​CAG​GTG​AAG​AA	TCA​CGT​AGG​CTT​CTA​TGC​AGT
PDE4B	CTG​GTA​CTT​CAT​GCC​GCC​TT	TGA​CAA​TCA​GGT​CAT​CGC​CG
β-actin	GGA​GAT​TAC​TGC​CCT​GGC​TCC​TA	GAC​TCA​TCG​TAC​TCC​TGC​TTG​CTG

### 2.11 Statistical analysis

All the data in this study were analyzed *via* GraphPad Prism (version 8.0.2) and are expressed as the means ± SEMs. Differences between two groups were analyzed *via* a *t*-test, and differences among multiple groups were detected *via* one-way ANOVA followed by Tukey’s *post hoc* test. Survival curves were plotted using Kaplan-Meier curves, and the statistical analysis of the survival curve was performed *via* the log-rank test. A p value < 0.05 was considered to indicate statistical significance.

## 3 Results

### 3.1 Rut relieves hepatic IRI in LT rats

**FIGURE 1:**
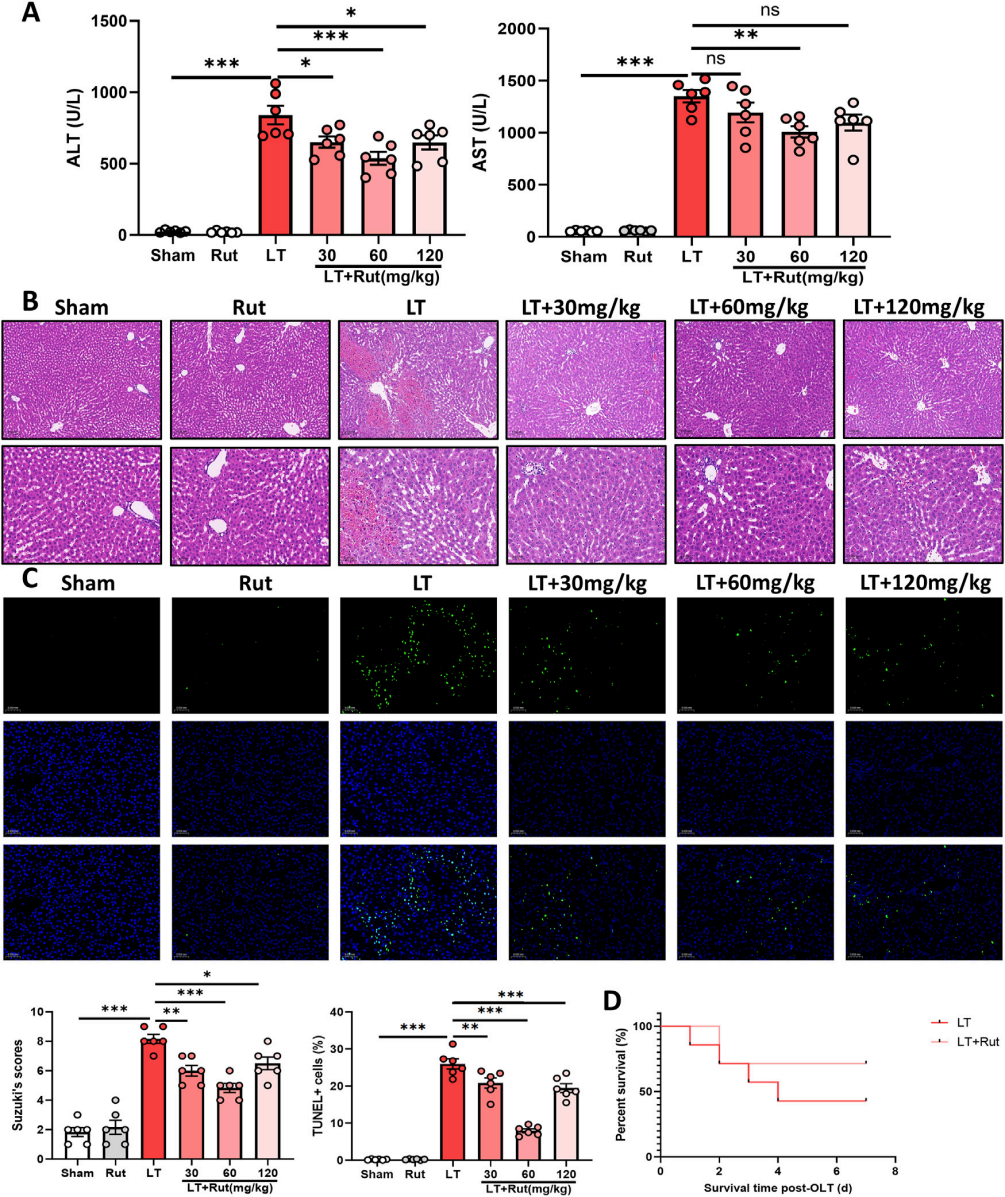
Rut relieves hepatic IRI in LT rats. **(A)** ALT and AST assays were performed to determine the effect of Rut on liver function after LT in rats. **(B)** H&E staining of liver tissues from rats after LT. **(C)** TUNEL staining of liver tissues from rats after LT. **(D)** Survival curves of the rats after LT. *P < 0.05; **P < 0.01; ***P < 0.001. ALT, alanine aminotransferase; AST, aspartate aminotransferase; IRI, ischemia‒reperfusion injury; Rut, rutaecarpine.

To explore the effect of Rut on hepatic IRI, we first constructed a DCD LT model in rats. As shown in Figure 1A, ALT and AST levels were significantly increased after LT, indicating impaired liver function. Rut treatment reduced ALT and AST levels and restored liver function in a dose-dependent manner. H&E staining revealed significant pathological alterations following LT (Figure 1B). There was notable swelling and degeneration of hepatocytes and liver sinusoidal endothelial cells. Furthermore, the hepatic cord structure exhibited disorganization, accompanied by congestion of central veins and sinusoids within the hepatic lobules. Stenosis of the liver sinusoids and extensive necrosis of the hepatic lobules were also observed. Rut treatment significantly reversed these changes, resulting in significantly lower Suzuki scores. In addition, Rut treatment inhibited the cell apoptosis induced by LT (Figure 1C). Finally, we observed the effect of Rut treatment on the survival rate of the rats. Rut treatment improved the survival rate from 43% to 71% (Figure 1D), but the difference was not statistically significant. Together, these results indicate that Rut can relieve IRI induced by LT.

### 3.2 Rut inhibits inflammation and oxidative stress in LT rats

**FIGURE 2:**
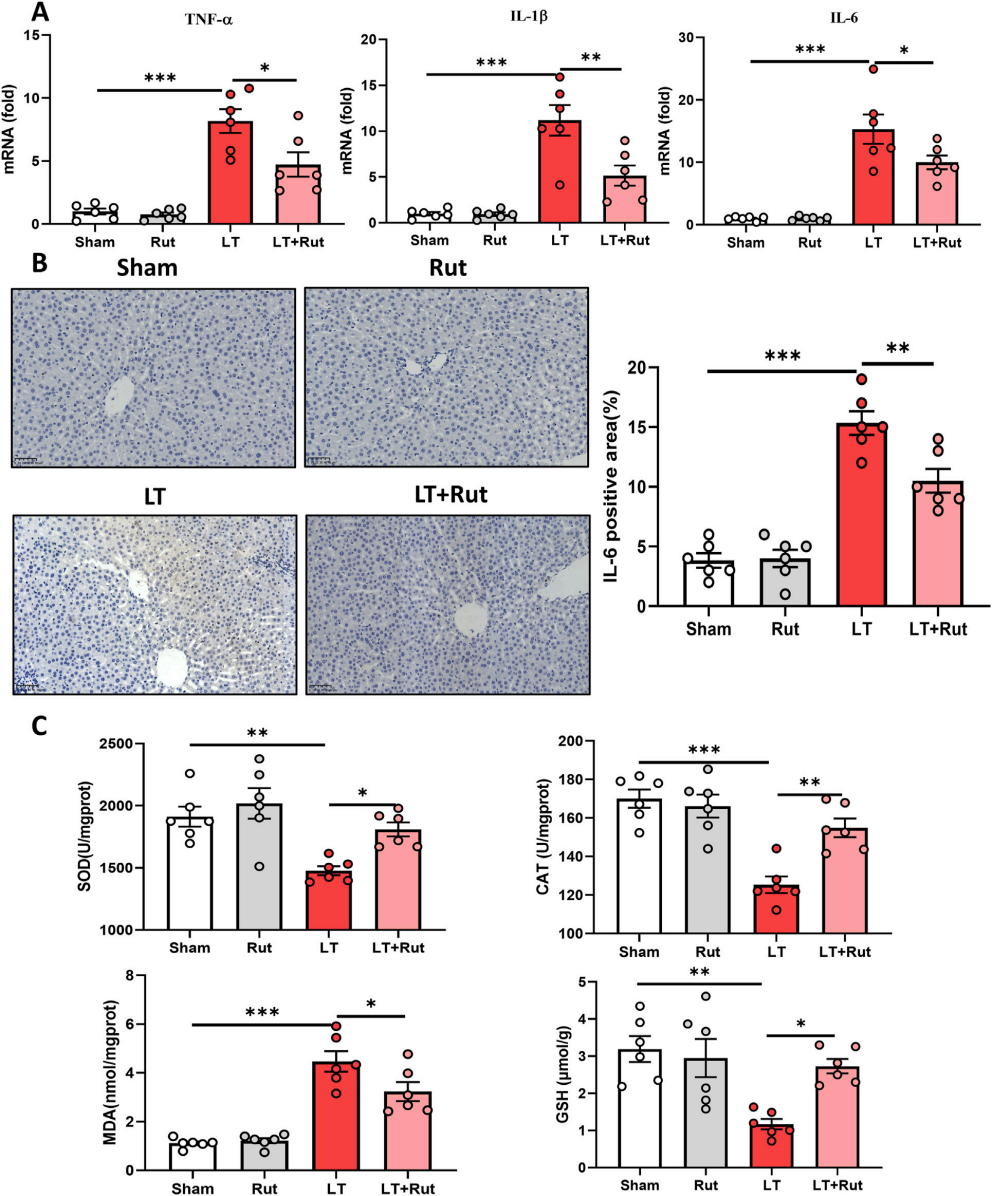
Rut inhibits inflammation and oxidative stress in LT rats. **(A)** RT‒PCR analysis of TNF-α, IL-1β, and IL6 to determine the effect of Rut on inflammation after LT in rats. **(B)** IHC staining of IL-6 in rat liver tissues after LT. **(C)** SOD, CAT, MDA and GSH assays in rat liver tissues after LT. *P < 0.05; **P < 0.01; ***P < 0.001. CAT, catalase; GSH, glutathione; LT, liver transplantation; MDA, malondialdehyde; Rut, rutaecarpine; SOD, superoxide dismutase.

We observed the effect of Rut treatment on inflammation by detecting the expression of proinflammatory cytokines. After LT, the expression of IL6, IL-1β, and TNF-α significantly increased, and Rut treatment significantly inhibited the expression of these inflammatory cytokines (Figures 2A,B). We further detected the activities of the antioxidant enzymes SOD and CAT as well as the levels of GSH. Antioxidant enzymes can eliminate reactive oxygen species (ROS) and inhibit oxidative stress. The results showed that after LT, the activities of SOD and CAT and the content of GSH significantly decreased, and the level of the oxidative stress product MDA increased (Figure 2C). After Rut treatment, the antioxidant enzyme system was partially restored, and the MDA reduced. Taken together, these findings suggest that Rut inhibits inflammation and oxidative stress induced by LT in rats.

### 3.3 Rut treatment attenuates OGD/R-induced cell damage, inflammation and oxidative stress

**FIGURE 3:**
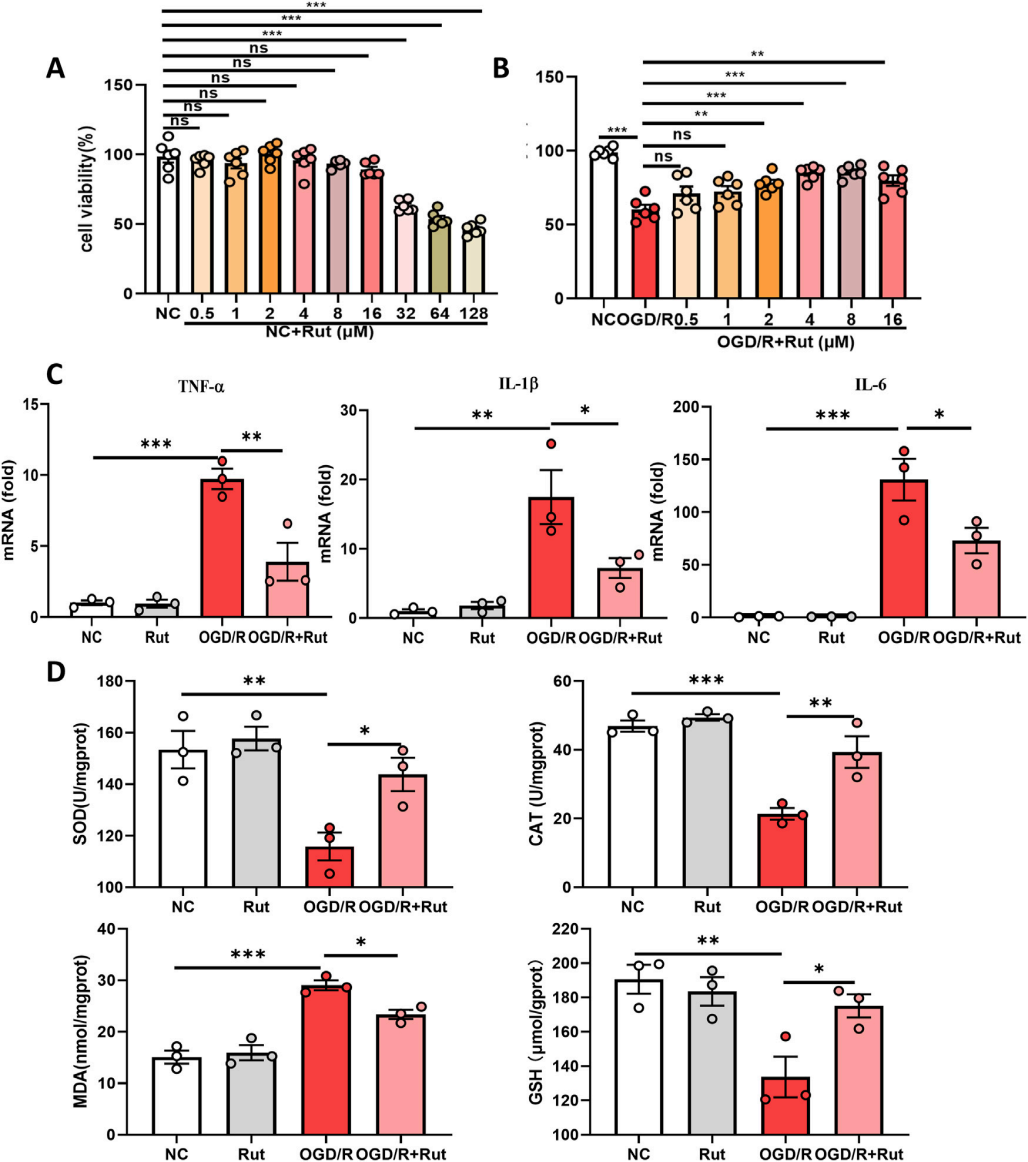
Rut treatment attenuates OGD/R-induced cell damage, inflammation and oxidative stress. **(A)** CCK-8 assay to detect the cytotoxicity of Rut on BRL-3A cells. **(B)** CCK-8 assay to determine the effect of Rut on OGD/R-induced BRL-3A cell damage. **(C)** RT‒PCR analysis of TNF-α, IL-1β, and IL6 in OGD/R-induced BRL-3A cells. **(D)** SOD, CAT, MDA and GSH assays in OGD/R-induced BRL-3A cells. *P < 0.05; **P < 0.01; ***P < 0.001. CAT, catalase; GSH, glutathione; OGD/R, oxygen‒glucose deprivation/reoxygenation; MDA, malondialdehyde; Rut, rutaecarpine; SOD, superoxide dismutase.

We also constructed an OGD/R model in rat hepatocytes to observe the effect of Rut in an *in vitro* model of hepatic IRI. First, we detected the cytotoxicity of Rut. The CCK-8 results revealed that when the Rut concentration was greater than 16 μM, Rut was toxic to hepatocytes (Figure 3A). We then explored the effect of Rut on OGD/R-induced cell damage and found that Rut dose-dependently attenuated OGD/R-induced cell damage and restored cell viability (Figure 3B). The protective effect was the strongest when the Rut concentration was four or 8 μM. Therefore, we chose Rut at a concentration of 4 μM for subsequent experiments. We detected inflammatory cytokines, and the RT‒PCR results showed that Rut treatment inhibited the expression of IL6, IL-1β, and TNF-α (Figure 3C). In addition, we detected the level of oxidative stress. Compared with OGD/R alone, Rut treatment increased the activities of SOD and CAT as well as the content of GSH and decreased the content of MDA (Figure 3D). Collectively, these results indicate that Rut treatment can attenuate OGD/R-induced cell damage, inflammation and oxidative stress.

### 3.4 PDE4B is the target of rut

**FIGURE 4:**
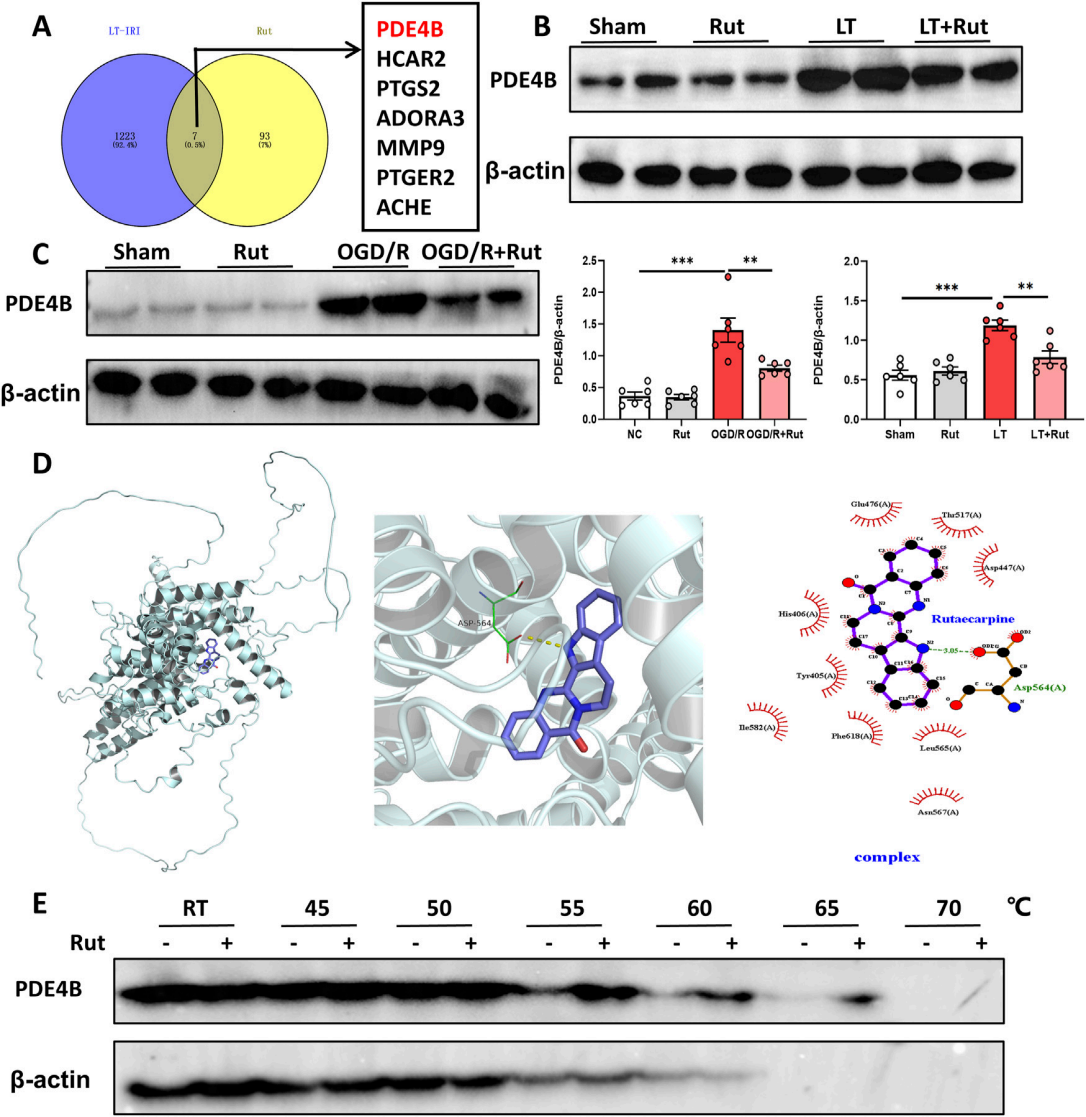
PDE4B is the target of Rut. **(A)** Venn diagram of the intersection between target genes of Rut and the differential genes of LT. **(B)** Western blot analysis of PDE4B in rat liver tissues after LT. **(C)** Western blot analysis of PDE4B in OGD/R-induced BRL-3A cells. **(D)** Molecular docking between Rut and PDE4B. **(E)** CETSA was used to verify that Rut directly targets PDE4B in BRL-3A cells. *P < 0.05; **P < 0.01; ***P < 0.001. CETSA, cellular thermal shift assay; OGD/R, oxygen‒glucose deprivation/reoxygenation; Rut, rutaecarpine.

To identify targets by which Rut exerts its hepatoprotective effect, we first analyzed the LT dataset (GSE151648) to obtain the differentially expressed genes between before and after reperfusion, that is, the genes that may play a role in hepatic IRI. The potential targets of Rut were obtained from the Swiss Target Prediction online database (http://www.swisstargetprediction.ch/), and we subsequently took the intersection of the obtained targets and the differential genes. A total of seven candidate targets were obtained, all of which are potential targets for the Rut to exert hepatoprotective effects (Figure 4A). Among them, PDE4B inhibition has been reported to reduce inflammation and oxidative stress in many studies (Blauvelt et al., 2023; Chen et al., 2024; Su et al., 2022), so we focused on PDE4B. In the LT and OGD/R models, PDE4B protein expression significantly increased (Figures 4B,C), indicating that PDE4B may play a role in the development of hepatic IRI. Rut treatment significantly decreased the PDE4B protein level. To further verify whether Rut directly targets PDE4B, we performed molecular docking and cellular thermal shift assay (CESTA). Rut and PDE4B formed hydrogen bonds and salt bridges (Figure 4D), proving that they are well combined. CETSA is a technology used to test intracellular drug (ligand) and protein (target) interactions. When a protein binds to a drug, its thermal stability (not stability) changes, and the interaction between the drug and protein can be identified by measuring such a change. CESTA results showed that Rut treatment enhanced the thermal stability of PDE4B (Figure 4E), further indicating that Rut directly targets PDE4B. Together, the above results indicate that PDE4B is the target of Rut in LT.

### 3.5 PDE4B regulates hepatic IRI

**FIGURE 5:**
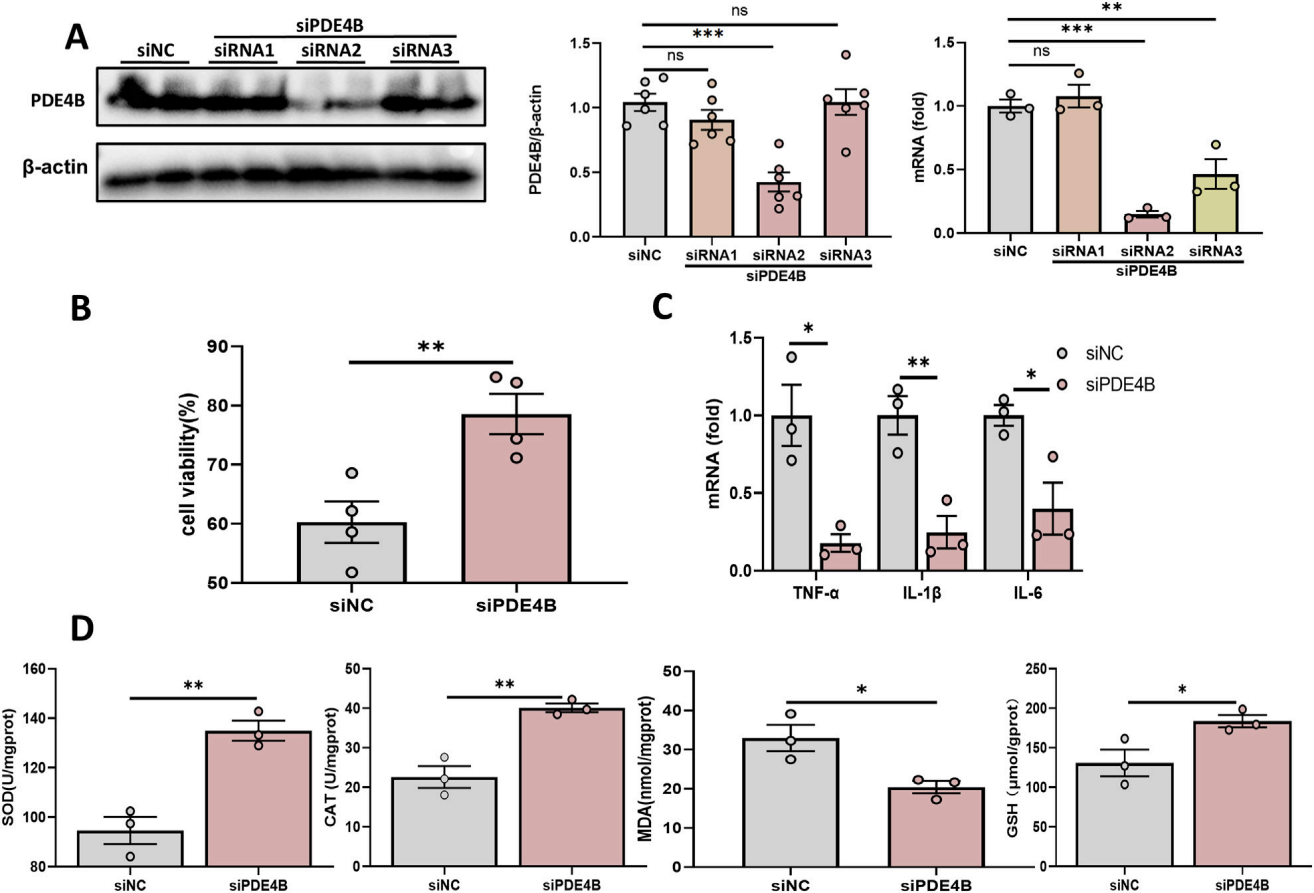
Inhibition of PDE4B alleviates hepatic IRI. **(A)** Validation of the PDE4B silencing efficiency in BRL-3A cells. **(B)** CCK-8 assay to determine the effect of PDE4B silencing on OGD/R-induced BRL-3A cell damage. **(C)** RT‒PCR analysis of TNF-α, IL-1β, and IL6 to determine the effect of PDE4B silencing on inflammation in OGD/R-induced BRL-3A cells. **(D)** SOD, CAT, MDA and GSH assays were performed to determine the effects of PDE4B silencing on oxidative stress in OGD/R-induced BRL-3A cells. *P < 0.05; **P < 0.01; ***P < 0.001. IRI, ischemia‒reperfusion injury; CAT, catalase; GSH, glutathione; MDA, malondialdehyde; OGD/R, oxygen‒glucose deprivation/reoxygenation; Rut, rutaecarpine; SOD, superoxide dismutase.

**FIGURE 6:**
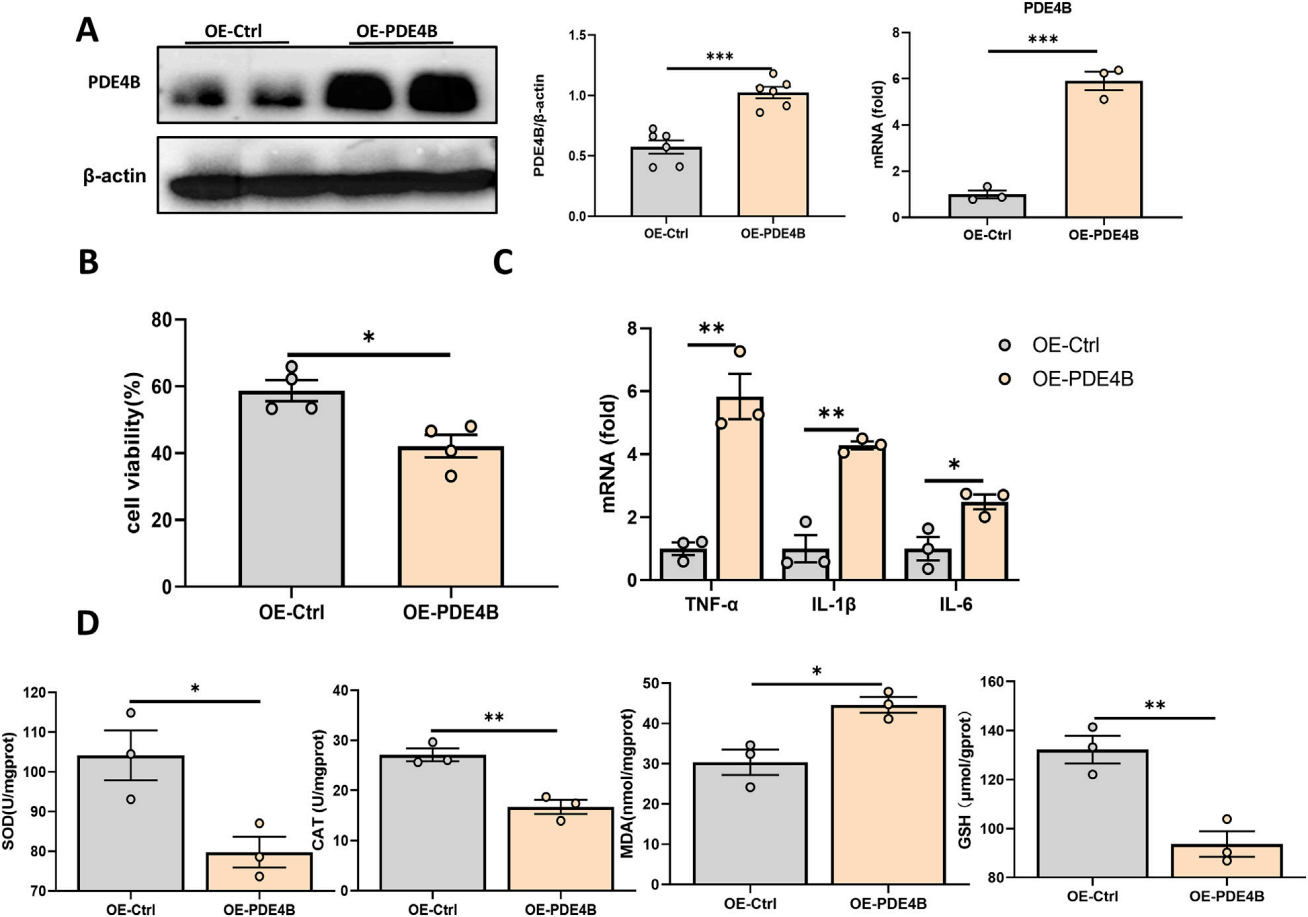
PDE4B overexpression aggravates hepatic IRI. **(A)** Validation of the PDE4B overexpression efficiency in BRL-3A cells. **(B)** CCK-8 assay to determine the effect of PDE4B overexpression on OGD/R-induced BRL-3A cell damage. **(C)** RT‒PCR analysis of TNF-α, IL-1β, and IL6 to determine the effect of PDE4B overexpression on inflammation in OGD/R-induced BRL-3A cells. **(D)** SOD, CAT, MDA and GSH assays were performed to determine the effects of PDE4B overexpression on oxidative stress in OGD/R-induced BRL-3A cells. *P < 0.05; **P < 0.01; ***P < 0.001. IRI, ischemia‒reperfusion injury; CAT, catalase; GSH, glutathione; MDA, malondialdehyde; OGD/R, oxygen‒glucose deprivation/reoxygenation; Rut, rutaecarpine; SOD, superoxide dismutase.

To elucidate the function of PDE4B in hepatic IRI, we silenced PDE4B in BRL-3A cells. WB and RT‒PCR confirmed that PDE4B was effectively silenced (Figure 5A). CCK8 assays revealed that PDE4B silencing significantly reversed the decrease in cell viability induced by OGD/R (Figure 5B). The results of RT‒PCR showed that PDE4B silencing inhibited the expression of the inflammatory cytokines IL6, IL-1β and TNF-α (Figure 5C). Furthermore, silencing PDE4B resulted in a significant enhancement of SOD and CAT activities, along with an increase in GSH levels, while concurrently reducing MDA content (Figure 5D). Conversely, overexpression of PDE4B exacerbated OGD/R-induced cellular damage and upregulated the expression of inflammatory cytokines IL-6, IL-1β, and TNF-α (Figures 6A–C). Following PDE4B overexpression, there was a further decline in the activities of SOD and CAT, as well as in GSH levels (Figure 6D). In summary, these results indicate that PDE4B aggravates hepatic IRI and that the inhibition of PDE4B can alleviate hepatic IRI.

### 3.6 Rut alleviates hepatic IRI in a PDE4B-dependent manner

**FIGURE 7:**
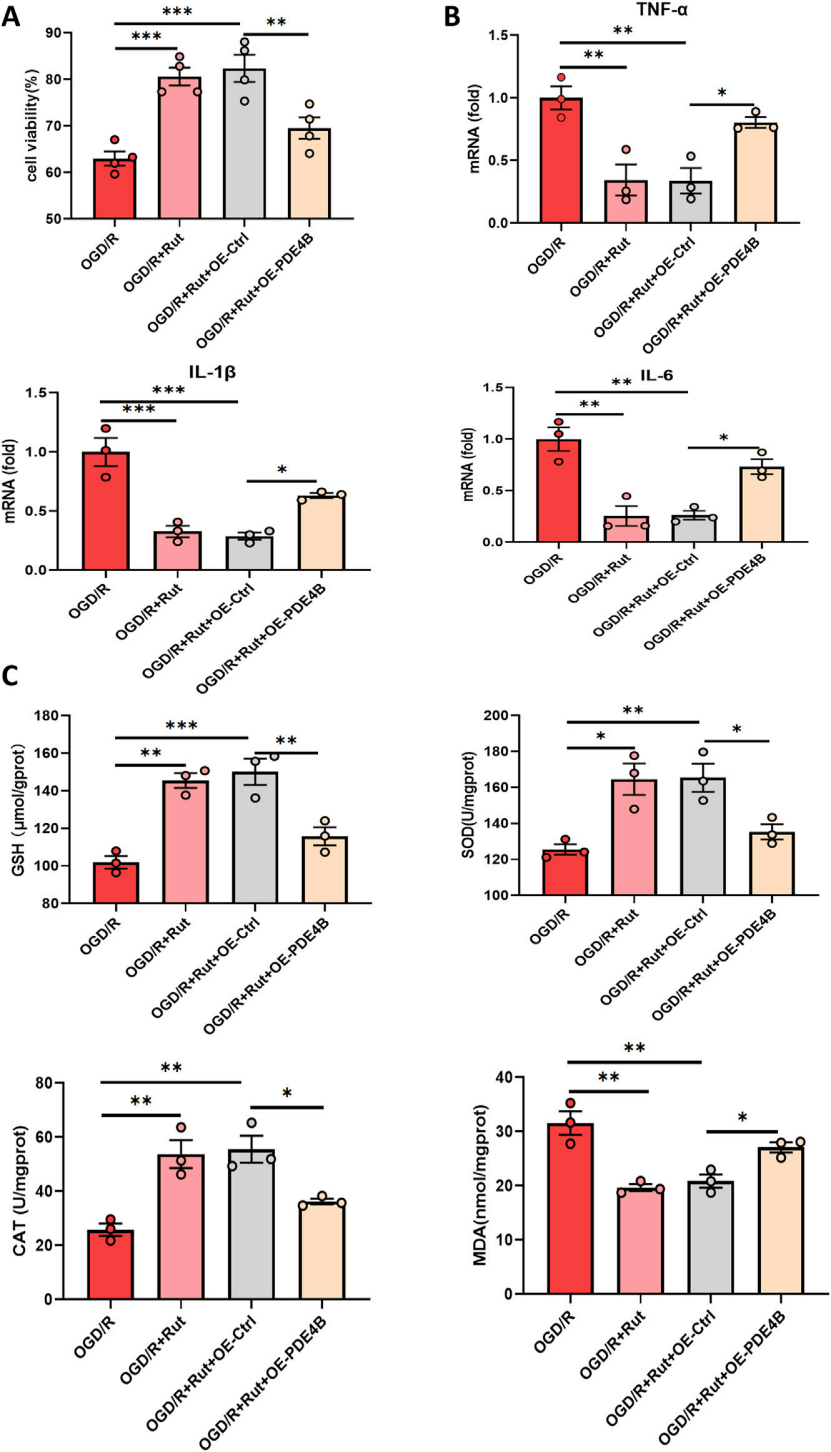
Rut alleviates hepatic IRI in a PDE4B-dependent manner. **(A)** A CCK-8 assay was used to determine whether the hepatoprotective effect of Rut is dependent on PDE4B. **(B)** RT‒PCR analysis of TNF-α, IL-1β, and IL6 to detect inflammation levels in OGD/R-induced BRL-3A cells. **(C)** SOD, CAT, MDA and GSH assays were performed to detect oxidative stress levels in OGD/R-induced BRL-3A cells. *P < 0.05; **P < 0.01; ***P < 0.001. CAT, catalase; GSH, glutathione; MDA, malondialdehyde; Rut, rutaecarpine; SOD, superoxide dismutase.

To determine whether the hepatoprotective effect of Rut on hepatic IRI is dependent on PDE4B inhibition, we overexpressed PDE4B in the Rut-treated group. The CCK8 assay demonstrated that PDE4B overexpression abolished the ability of Rut treatment to alleviate OGD/R-induced cell damage (Figure 7A), and PDE4B overexpression counteracted the decreases in the expression levels of the inflammatory cytokines IL6, IL-1β, and TNF-α (Figure 7B). Similarly, PDE4B overexpression diminished the efficacy of Rut treatment to increase the levels of SOD and GSH and the activity of CAT (Figure 7C). These findings suggest that the alleviation of hepatic IRI by Rut is closely related to the inhibition of PDE4B.

## 4 Discussion

To the best of our knowledge, this is the first study to apply Rut in LT. Using *in vivo* and *in vitro* models, we found that Rut can reduce hepatic IRI through the inhibition of inflammation and oxidative stress. Further mechanistic studies confirmed that PDE4B is a key target through which Rut exerts its hepatoprotective effect. These results emphasize the possibility of the application of Rut in patients undergoing LT.

IRI severely limits donor source and the efficacy of LT. Non-ischemic LT can avoid IRI to a certain extent and is an innovative milestone in the organ transplantation field (Guo et al., 2023). However, DCD livers are inevitably exposed to a longer warm ischemia period. Therefore, DCD livers are more likely to develop IRI and are considered extended-criteria donor livers. A large number of DCD livers were discarded because of concerns about the outcome after transplantation (Thuong et al., 2016). How to relieve hepatic IRI and increase the DCD livers utilization has always been a difficult problem, and various teams around the world are committed to solving this problem. Traditional Chinese medicine emphasizes overall regulation and has multi-component, multi-target characteristics. We previously demonstrated that *Scutellaria baicalensis Georgi* can relieve hepatic IRI in LT through multi-targets (Liu et al., 2024). However, the multi-target characteristics also limit further breakthroughs in traditional Chinese medicine research. Chinese medicine monomers have clear chemical compositions, facilitating the identification of direct functional targets and enhancing the feasibility of large-scale production. Rut is the active ingredient of Evodia rutaecarpa and has been confirmed to have hepatoprotective effects on various liver diseases (Li et al., 2020). However, it has not been used in IRI induced by LT. Our studies confirmed that Rut can restore liver function, reduce pathological damage, inhibit apoptosis, and improve the prognosis of LT. Given that Rut has been used in clinical practice for many years, it is relatively feasible to use Rut in LT patients (Warner et al., 2004).

Hepatic IRI is considered a local sterile inflammatory response driven by innate immunity (Ahmed et al., 2021). Warm and cold ischemic injury and subsequent reperfusion trigger hepatocytes to release damage-associated molecular patterns, which induce an inflammatory cascade and further aggravate hepatocyte injury (Zhou et al., 2016). Cytokines such as IL6, IL-1β and TNF-α can activate Kupffer cells and neutrophils to trigger an intense cytotoxic immune response and play a key pathogenic role in the entire pathophysiological process of hepatic IRI (Kaltenmeier et al., 2022). Rut can inhibit the production of these proinflammatory factors *in vivo* and *in vitro*, resulting in inhibiting inflammation. Oxidative stress is also an important driving factor in the pathogenesis of hepatic IRI. Excessive ROS and the consumption of endogenous antioxidants cause redox imbalance, which leads to oxidative stress and further triggers apoptosis and necrotic cell death (G Bardallo et al., 2022). Endogenous antioxidant enzymes, such as SOD, CAT, and GSH, play crucial roles in scavenging ROS and thereby effectively mitigating oxidative stress in hepatic tissues (Romani et al., 1988; Yan et al., 2022). In our study, Rut effectively increased the levels of antioxidant enzymes while decreasing the levels of the oxidative stress product MDA. These results support the anti-inflammatory and anti-oxidative stress activity of Rut.

To further explore the downstream targets of Rut in hepatic IRI, we analyzed LT datasets before and after reperfusion and the downstream targets of the Rut. PDE4B was found to be the downstream target of Rut to exert the hepatoprotective effects. We used molecular docking and CESTA to further clarify the direct binding between Rut and PDE4B. PDE4B is an isoform of PDE4 that mainly depends on the hydrolysis of cAMP to exert its biological effects. Inhibition of PDE4 isoforms increases intracellular cyclic adenosine monophosphate levels and suppresses inflammation by reducing the release of proinflammatory mediators and the recruitment of inflammatory cells (Maurice et al., 2014; Zuo et al., 2019). PDE4B knockout mice do not even develop airway inflammation (Jin et al., 2010). PDE4B deletion or inhibition can relieve neutrophil inflammation and thus myocardial IRI (Wan et al., 2022). Meanwhile, PDE4B inhibition can enhance the nuclear translocation of nuclear factor erythroid 2-related factor 2 (Nrf-2), thereby reducing oxidative stress (Xu et al., 2020; Xu et al., 2021). However, the role of PDE4B in hepatic IRI remains unclear. In this study, PDE4B was highly expressed in rats that experienced LT as well as in OGD/R-treated BRL-3A cells. PDE4B knockdown significantly improved cell activity and inhibited inflammation and oxidative stress. The overexpression of PDE4B was found to suppress cellular activity while exacerbating inflammation and oxidative stress. These results suggest that PDE4B can aggravate hepatic IRI and thus represents a potential target for therapeutic intervention in the management of hepatic IRI. In addition, the overexpression of PDE4B abolished the effects of Rut on OGD/R-induced cell damage, inflammation and oxidative stress. These results indicate that the ability of Rut to relieve hepatic IRI is dependent on PDE4B. We did not further explore how PDE4B regulates inflammation and oxidative stress, which is a weakness of this study, although this has been explained to some extent in other disease models.

This study has several other limitations. First, we explored the role of only one target, PDE4B, and no further verification was conducted on other targets of Rut. Although Rut has been shown to play a hepatoprotective role through PDE4B, thorough target validation will facilitate a more complete understanding of how Rut works. Second, only three doses of Rut were selected, and a dose‒response curve of Rut in LT needs to be drawn to find the optimal timing and dose for Rut administration. It also needs to be determined whether multiple doses before transplantation are more effective.

## 5 Conclusion

In summary, through *in vivo* and *in vitro* models, we demonstrated that Rut has hepatoprotective effects against IRI induced by LT through its anti-oxidant and anti-inflammatory activities. PDE4B aggravates hepatic IRI and is the target of Rut, and inhibition of PDE4B can alleviate hepatic IRI. More in-depth studies on the mechanism of Rut in LT will be beneficial for the clinical application of Rut.

## Data Availability

The raw data supporting the conclusions of this article will be made available by the authors, without undue reservation.

## References

[B1] Ahmed O. Robinson M. W. O'Farrelly C. (2021). Inflammatory processes in the liver: divergent roles in homeostasis and pathology. Cell. Mol. Immunol. 18, 1375–1386. 10.1038/s41423-021-00639-2 33864004 PMC8166849

[B2] Bao M. H. Dai W. Li Y. J. Hu C. P. (2011). Rutaecarpine prevents hypoxia-reoxygenation-induced myocardial cell apoptosis via inhibition of NADPH oxidases. Can. J. Physiol. Pharmacol. 89, 177–186. 10.1139/Y11-006 21423291

[B3] Blauvelt A. Langley R. G. Gordon K. B. Silverberg J. I. Eyerich K. Sommer M. O. A. (2023). Next generation PDE4 inhibitors that selectively target PDE4B/D subtypes: a narrative review. Dermatol Ther. (Heidelb) 13, 3031–3042. 10.1007/s13555-023-01054-3 37924462 PMC10689637

[B4] Chen K. Xu B. Long L. Wen H. Zhao Q. Tu X. (2024). Inhibition of phosphodiesterase 4 suppresses neuronal ferroptosis after cerebral ischemia/reperfusion. Mol. Neurobiol. 10.1007/s12035-024-04495-9 39287745

[B5] Chun K. S. Kim E. H. Kim D. H. Song N. Y. Kim W. Na H. K. (2024). Targeting cyclooxygenase-2 for chemoprevention of inflammation-associated intestinal carcinogenesis: an update. Biochem. Pharmacol. 228, 116259. 10.1016/j.bcp.2024.116259 38705538

[B6] Dar W. A. Sullivan E. Bynon J. S. Eltzschig H. Ju C. (2019). Ischaemia reperfusion injury in liver transplantation: cellular and molecular mechanisms. Liver Int. 39, 788–801. 10.1111/liv.14091 30843314 PMC6483869

[B7] Estari R. K. Dong J. Chan W. K. Park M. S. Zhou Z. (2021). Time effect of rutaecarpine on caffeine pharmacokinetics in rats. Biochem. Biophys. Rep. 28, 101121. 10.1016/j.bbrep.2021.101121 34527815 PMC8429912

[B8] G Bardallo G. B. Panisello-Rosello A. Sanchez-Nuno S. Alva N. Rosello-Catafau J. Carbonell T. (2022). Nrf2 and oxidative stress in liver ischemia/reperfusion injury. FEBS J. 289, 5463–5479. 10.1111/febs.16336 34967991

[B9] Guo Z. Zhao Q. Jia Z. Huang C. Wang D. Ju W. (2023). A randomized-controlled trial of ischemia-free liver transplantation for end-stage liver disease. J. Hepatol. 79, 394–402. 10.1016/j.jhep.2023.04.010 37086919

[B10] Hashimoto K. (2020). Liver graft from donation after circulatory death donor: real practice to improve graft viability. Clin. Mol. Hepatol. 26, 401–410. 10.3350/cmh.2020.0072 32646199 PMC7641554

[B11] Jia D. Wu K. Luo J. Xu X. Pan W. Zhao M. (2024). Wogonin alleviates DCD liver ischemia/reperfusion injury by regulating ALOX15/iNOS-mediated ferroptosis. Transplantation 108, 2374–2385. 10.1097/TP.0000000000005123 38946036

[B12] Jin S. L. Goya S. Nakae S. Wang D. Bruss M. Hou C. (2010). Phosphodiesterase 4B is essential for T(H)2-cell function and development of airway hyperresponsiveness in allergic asthma. J. Allergy Clin. Immunol. 126, 1252–1259.e12. 10.1016/j.jaci.2010.08.014 21047676 PMC3002752

[B13] Jin S. W. Hwang Y. P. Choi C. Y. Kim H. G. Kim S. J. Kim Y. (2017). Protective effect of rutaecarpine against t-BHP-induced hepatotoxicity by upregulating antioxidant enzymes via the CaMKII-Akt and Nrf2/ARE pathways. Food Chem. Toxicol. 100, 138–148. 10.1016/j.fct.2016.12.031 28025122

[B14] Kaltenmeier C. Wang R. Popp B. Geller D. Tohme S. Yazdani H. O. (2022). Role of immuno-inflammatory signals in liver ischemia-reperfusion injury. Cells 11, 2222. 10.3390/cells11142222 35883665 PMC9323912

[B15] Ko H. C. Tsai T. H. Chou C. J. Hsu S. Y. Li S. Y. Chen C. F. (1994). High-performance liquid chromatographic determination of rutaecarpine in rat plasma: application to a pharmacokinetic study. J. Chromatogr. B Biomed. Appl. 655, 27–31. 10.1016/s0378-4347(94)80128-2 8061830

[B16] Kshirsagar U. A. (2015). Recent developments in the chemistry of quinazolinone alkaloids. Org. Biomol. Chem. 13, 9336–9352. 10.1039/c5ob01379h 26278395

[B17] Li X. Ge J. Zheng Q. Zhang J. Sun R. Liu R. (2020). Evodiamine and rutaecarpine from Tetradium ruticarpum in the treatment of liver diseases. Phytomedicine 68, 153180. 10.1016/j.phymed.2020.153180 32092638

[B18] Liu J. Guo S. Gong J. Cheng L. Luo J. Cheng M. (2024). Identification of the anti-ischemia-reperfusion injury effect of baicalein from Scutellaria baicalensis Georgi in liver transplantation. Curr. Med. Chem. 31. 10.2174/0109298673304206240702062057 38988155

[B19] Masior L. Grat M. (2022). Primary nonfunction and early allograft dysfunction after liver transplantation. Dig. Dis. 40, 766–776. 10.1159/000522052 35114676

[B20] Maurice D. H. Ke H. Ahmad F. Wang Y. Chung J. Manganiello V. C. (2014). Advances in targeting cyclic nucleotide phosphodiesterases. Nat. Rev. Drug Discov. 13, 290–314. 10.1038/nrd4228 24687066 PMC4155750

[B21] Meier R. P. H. Nunez M. Syed S. M. Feng S. Tavakol M. Freise C. E. (2023). DCD liver transplant in patients with a MELD over 35. Front. Immunol. 14, 1246867. 10.3389/fimmu.2023.1246867 37731493 PMC10507358

[B22] Moon T. C. Murakami M. Kudo I. Son K. H. Kim H. P. Kang S. S. (1999). A new class of COX-2 inhibitor, rutaecarpine from Evodia rutaecarpa. Inflamm. Res. 48, 621–625. 10.1007/s000110050512 10669112

[B23] Romani F. Vertemati M. Frangi M. Aseni P. Monti R. Codeghini A. (1988). Effect of superoxide dismutase on liver ischemia-reperfusion injury in the rat: a biochemical monitoring. Eur. Surg. Res. 20, 335–340. 10.1159/000128783 3224631

[B24] Schwab S. Elmer A. Sidler D. Straumann L. Sturzinger U. Immer F. (2024). Selection bias in reporting of median waiting times in organ transplantation. JAMA Netw. Open 7, e2432415. 10.1001/jamanetworkopen.2024.32415 39254975 PMC11388028

[B25] Su Y. Ding J. Yang F. He C. Xu Y. Zhu X. (2022). The regulatory role of PDE4B in the progression of inflammatory function study. Front. Pharmacol. 13, 982130. 10.3389/fphar.2022.982130 36278172 PMC9582262

[B26] Thuong M. Ruiz A. Evrard P. Kuiper M. Boffa C. Akhtar M. Z. (2016). New classification of donation after circulatory death donors definitions and terminology. Transpl. Int. 29, 749–759. 10.1111/tri.12776 26991858

[B27] Wan Q. Xu C. Zhu L. Zhang Y. Peng Z. Chen H. (2022). Targeting PDE4B (Phosphodiesterase-4 subtype B) for cardioprotection in acute myocardial infarction via neutrophils and microcirculation. Circ. Res. 131, 442–455. 10.1161/CIRCRESAHA.122.321365 35899614

[B28] Warner D. S. Sheng H. Batinic-Haberle I. (2004). Oxidants, antioxidants and the ischemic brain. J. Exp. Biol. 207, 3221–3231. 10.1242/jeb.01022 15299043

[B29] Wu X. Y. Zhao M. J. Liao W. Liu T. Liu J. Y. Gong J. H. (2024). Oridonin attenuates liver ischemia-reperfusion injury by suppressing PKM2/NLRP3-mediated macrophage pyroptosis. Cell. Immunol. 401-402, 104838. 10.1016/j.cellimm.2024.104838 38810591

[B30] Xu B. Qin Y. Li D. Cai N. Wu J. Jiang L. (2020). Inhibition of PDE4 protects neurons against oxygen-glucose deprivation-induced endoplasmic reticulum stress through activation of the Nrf-2/HO-1 pathway. Redox Biol. 28, 101342. 10.1016/j.redox.2019.101342 31639651 PMC6807264

[B31] Xu B. Xu J. Cai N. Li M. Liu L. Qin Y. (2021). Roflumilast prevents ischemic stroke-induced neuronal damage by restricting GSK3β-mediated oxidative stress and IRE1α/TRAF2/JNK pathway. Free Radic. Biol. Med. 163, 281–296. 10.1016/j.freeradbiomed.2020.12.018 33359910

[B32] Yan R. Ren J. Wen J. Cao Z. Wu D. Qin M. (2022). Enzyme therapeutic for ischemia and reperfusion injury in organ transplantation. Adv. Mater 34, e2105670. 10.1002/adma.202105670 34617335

[B33] Yang B. Dong Y. Xu Z. Li X. Wang F. Zhang Y. (2022). Improved stability and pharmacokinetics of wogonin through loading into PASylated ferritin. Colloids Surf. B Biointerfaces 216, 112515. 10.1016/j.colsurfb.2022.112515 35512464

[B34] Zhai Y. Petrowsky H. Hong J. C. Busuttil R. W. Kupiec-Weglinski J. W. (2013). Ischaemia-reperfusion injury in liver transplantation--from bench to bedside. Nat. Rev. Gastroenterol. Hepatol. 10, 79–89. 10.1038/nrgastro.2012.225 23229329 PMC3577927

[B35] Zhang Y. Wang S. Dai M. Nai J. Zhu L. Sheng H. (2020). Solubility and bioavailability enhancement of oridonin: a review. Molecules 25, 332. 10.3390/molecules25020332 31947574 PMC7024198

[B36] Zhou Z. Xu M. J. Gao B. (2016). Hepatocytes: a key cell type for innate immunity. Cell. Mol. Immunol. 13, 301–315. 10.1038/cmi.2015.97 26685902 PMC4856808

[B37] Zuo H. Cattani-Cavalieri I. Musheshe N. Nikolaev V. O. Schmidt M. (2019). Phosphodiesterases as therapeutic targets for respiratory diseases. Pharmacol. Ther. 197, 225–242. 10.1016/j.pharmthera.2019.02.002 30759374

